# A community-based health–social partnership program for community-dwelling older adults: a hybrid effectiveness–implementation pilot study

**DOI:** 10.1186/s12877-022-03463-z

**Published:** 2022-10-07

**Authors:** Arkers Kwan Ching Wong, Frances Kam Yuet Wong, Martin Chi Sang Wong, Karen Kit Sum Chow, Dilys Kwai Sin Kwan, Dubby Yun Sang Lau

**Affiliations:** 1 School of Nursing, The Hong Kong Polytechnic University, 1 Cheong Wan Road, Hung Hom, Hong Kong; 2 The Jockey Club School of Public Health and Primary Care, Faculty of Medicine, the Chinese University of Hong Kong, Shatin, Hong Kong; 3 The Hong Kong Lutheran Social Service, Homantin, Hong Kong

**Keywords:** Health–social, Hybrid effectiveness–implementation, Older adults, Self-care

## Abstract

**Background:**

A growing body of literature supports the efficacy of the health–social approach for the implementation of complex interventions to enhance self-care health management among community-dwelling older adults. However, there is little research on how interventions with this approach are implemented and disseminated in a real community setting.

**Methods:**

This pilot study adopted an effectiveness–implementation hybrid design to 1) evaluate the effectiveness of a community-based Health–Social Partnership Program (HSPP) and 2) explore the reach, adoption, implementation, and maintenance of the HSPP in the community. Potential participants were recruited if they were aged 60 or above, owned a smartphone, and were cognitively competent. The participants received nurse-led case management with support from a social service team. Factors that hindered or facilitated the program delivery were examined to determine the implementation outcomes and sustained effects of the program. Data were collected at pre-intervention (T1), immediately post-intervention (T2), and 3 months post-intervention (T3).

**Results:**

Ninety-two older adults joined and completed the program. The recruitment rate was 76.7%. A significant interaction effect was found for the mean self-efficacy scores from T1 to T2 (Wald χ^2^ = 12.28, *p* ≤ .001). Barriers to widespread program implementation included manpower shortage, lack of experienced staff, and unpredictable environment, whereas facilitators, as suggested by the older adults, providers, and community staff members, included regular communication between the research and service teams, recruitment of participants through community centers with the support of the research team, and seamless partnership among the health–social partnership team members. Strong implementation fidelity was achieved with zero attrition rate.

**Conclusion:**

Most conventional randomized controlled trials investigating the effects of community-based programs have tended to control the contextual factors rather than incorporate the program in a real setting. This pilot study was the first to use a hybrid model to test the effectiveness and outcomes of HSPP implementation. The results imply that the program has a high potential sustainability in the real-life context.

**Trial registration:**

This study was registered at clinicaltrials.gov (NCT04442867; date of first registration 23/06/2020).

**Supplementary Information:**

The online version contains supplementary material available at 10.1186/s12877-022-03463-z.

Contributions to the literature

- Implementing, sustaining, and scaling up health promotion programs for older adults in the community setting are challenging but imperative to improve public health outcomes.
- This study is the first to involve key stakeholders, including the managers and frontline staff members of a local community center, to contribute to the implementation of a self-care health management program for community-dwelling older adults.
- The findings provide preliminary information on the facilitators of and barriers to implementing and sustaining a health–social partnership program for older adults in the community.

## Background

The population of Hong Kong, as in most countries, is aging. It is predicted that by 2038, 31.9% of Hong Kong’s population will be over the age of 65 [1]. Older adults tend to use hospital services more frequently, accounting for more than half of the total hospital admissions [2]. They are more likely to seek help from acute-care facilities and be hospitalized because of not only their susceptibility to physical disabilities and chronic conditions but also the lack of focused health and social support services for them in the community. A scoping review highlighted that although many older adults would prefer to maintain their independence and stay in their homes as they age, lack of professional advice on self-care strategies, fragmentation of health and social services, and lack of information on community services may diminish their ability to do so [3].

Self-care is defined as individuals taking action by themselves to prevent disease, maintain and promote health and functioning, and manage chronic illnesses and disabilities [4, 5]. Evidence suggests that people who adhere to self-care activities have a lower rate of health service utilization and lower health-care costs than those who cannot [6, 7]. Self-care can also increase their life satisfaction [8], well-being [9], quality of life [10], and, most importantly, ability to age in place [11]. However, self-care is easier said than done, especially for aged individuals who face barriers such as lack of medication and disease management knowledge, motivation and confidence in taking care of themselves, physical strength, and social support [12, 13]. In addition, social determinants of health, such as low educational level, separation from a spouse, and advancing age may impair one’s ability to self-care since these factors affect a person’s capacity, opportunity, and engagement to make the healthy behavioral change [14, 15]. While informal caregivers such as family members, assume increasingly greater responsibility nowadays for making treatment decisions, performing daily tasks, and providing physical and social supports to their older relatives, they do not have sufficient confidence, knowledge, and skills to properly manage the complex health and social needs, and achieve overall better health outcomes of the older adults without the assistance of health and social care professionals [16].

Complex interventions have emerged as a strategy to promote and support self-care among older adults by targeting multidimensional aspects to bring about synergistic effects [17]. Complex interventions, which involve a combination of several interacting components, can support self-care in the target population when delivered through an integrated, person-centered care approach by a multidisciplinary team [17]. The interacting components generally include holistic health assessment, empowerment (interventions that allow older adults to gain mastery over self-care management [18]), self-efficacy enhancement (interventions that increase the self-confidence of managing one’s illness [19]), and referral services [20]. A Canadian study implemented a program involving comprehensive geriatric health assessment, care planning (referred to as the process by which health care professionals and older adults discuss, agree and review an action plan to achieve the goals or health behavior change [21]), and referral to and coordination of community health and social services for a group of independent community-dwelling older adults [22]. The intervention group showed greater improvement in quality of life and depressive symptoms than the control group. Another program provided multidimensional assessment and interdisciplinary care based on a tailor-made treatment plan for community-dwelling older adults to improve their self-care skills and knowledge [23]. The intervention group had better self-rated health and activities of daily living than the usual-care group. Building on these findings [20], our team recently implemented a program involving proactive, individualized care assessment and coordinated care using a nurse-led, health–social multidisciplinary team approach. The results demonstrated that the program was effective in improving self-efficacy, quality of life, medication adherence, and use of health services among community-dwelling older adults [24]. Studies have revealed that both nurses and social workers (SWs) play crucial roles in helping older adults address health and social issues and support their healthy living in the community. However, the care needs to be well coordinated among the multidisciplinary team members with seamless collaboration to prevent the duplication and fragmentation of services.

A growing body of literature supports the involvement of health–social partnership teams in delivering complex interventions for community-dwelling older adults to enhance their self-care health management, although important operational concerns remain to be addressed. Such intervention programs face a number of challenges to sustainability in the real-world setting. Possible implementation-related barriers include insufficient training of team members, lack of equipment, and poor commitment of the management and operational staff in the organizations. Inadequate human or financial resources and lack of clarity on operational guidelines may also result in interventions being completely different from those planned originally and thus not achieving the intended results [25]. It is therefore imperative to evaluate not only the effects of such programs but also their implementation fidelity and sustainability.

An effectiveness–implementation hybrid design can enable researchers to obtain information regarding the intervention effects as well as the facilitators of and barriers to implementation [26]. To the best of the authors’ knowledge, there is a paucity of research trials with this design evaluating self-care health promotion programs for community-dwelling older adults [27, 28]. This pilot study intended to address this gap by implementing a health–social partnership program (HSPP) in the community and evaluating both the effectiveness and implementation outcomes. The results may facilitate effective research translation and provide important information to inform policymakers of the best strategy to implement, deliver, and sustain health promotion programs in the community.

### Conceptual framework

The design of the HSPP intervention was informed by Ecological theory [29]. Ecological theory from Bronfenbrenner asserts that self-care is influenced by factors at three levels of the environment: microsystem, mesosystem, and macrosystem [29]. The microsystem level consists of modifiable personal factors such as self-efficacy. In order to improve the older adults’ self-efficacy, the four hierarchical sources of Bandura’s self-efficacy theory were employed in this study [19]. The mesosystem level focuses on the interrelationships among older adults and the persons who have close connections with them, such as health-care providers. The nurse in this study used the Omaha System to assess the older adults’ health condition holistically, empower them to set goals and follow action plans, and provide self-care education and information to build ongoing trusting relationship with older adults [30]. The macrosystem level is an extension of the mesosystem level, which involves cross-boundary relationships among the different organizations. Gittell’s relational coordination theory was adopted in this regard to bridge the gap and break the boundaries between different health and social care disciplines so that they can provide the best integrated care to older adults to age in place [31].

## Methods

### Study design

This pilot study adopted a hybrid design to evaluate both the effectiveness and implementation outcomes of a community-based HSPP. The HSPP effectiveness was evaluated using a randomized controlled trial design with individuals randomized to either the intervention or the control group. A mixed methods design was used to assess the HSPP implementation outcomes and describe, both qualitatively and quantitatively, the factors that influence the implementation process.

### Study setting, participants, and recruitment strategies

A community center from a non-governmental organization had agreed to participate in this pilot study. Individuals who were living in the service areas of the community center and were its members were screened and recruited into the study if they 1) were aged 60 or above, 2) owned a smartphone, and 3) were cognitively competent, defined as Hong Kong version of Montreal Cognitive Assessment (HK-MoCA) scores ≥22 [32]. Individuals were excluded if they were 1) not able to communicate; 2) not reachable by phone; 3) not living at home; 4) bedbound; 5) living in an area with no Internet coverage; 6) already engaged in a structured health or social program; or 7) not staying in Hong Kong during the program period. A trained research assistant recruited participants from a member list provided by the community center, obtained their consent to join the program, and helped collect their baseline data.

The participants were randomized 1:1 to the intervention and control groups. One of the co-investigators generated the random group assignments using Research Randomizer. The group assignments were sealed in envelopes and opened sequentially at the time of group allocation. The participants in the intervention group received the intervention from the nurse case manager (NCM), whereas those in the control group received monthly social calls from another trained assistant. The research assistant who collected the data was blinded to the group allocation, but the healthcare providers were not.

### Sample size

As this was a pilot study aimed at implementing and testing the feasibility of a self-care health management program in the community, we did not limit the number of participants. As a general rule for pilot studies, the minimum number of participants should be set at not less than 30 in each group [33]. As previous programs for community-dwelling older adults have reported a drop-out rate of 10–15%, here we assumed a drop-out rate of 20%. Accordingly, it was necessary to include at least 72 participants in this study.

### Ethical consideration

The study followed the Declaration of Helsinki and was approved by the ethics committee of a university in Hong Kong before the commencement of the program. Information on the significance, purposes, procedures, risks, and benefits of the study and program were provided to all eligible participants. All participants signed a written consent form after expressing that they had understood the study.

### Intervention group

#### The effectiveness part

In the 3-month HSPP, the first month was treated as an intensive loading dose, involving one Zoom meeting and one call by the NCM and two follow-up telephone calls by the community workers (CWs). The subsequent maintenance dose was less intensive, involving one Zoom meeting with a CW and one call by the NCM in the second month and one Zoom meeting with a CW and a closing Zoom meeting with the NCM in the third month (See Additional file 1).

The health–social care team was led by a registered nurse (the NCM) and included CWs, an SW, a traditional Chinese medicine practitioner (TCM), and a group of general practitioners who provide medical services to the residents of the district where the community center is located. The NCM was involved in the initial assessment of the participants using the Omaha system, a comprehensive and holistic assessment tool used to identify the needs and problems of an individual in four domains: environmental, psychosocial, physiological, and health-related behavior [30]. The Omaha system is applicable and valid for community-dwelling older adults in Hong Kong [34]. The team extracted the participants’ problems using the Omaha system and classified them into health, social, and health–social partnership-related problems. The NCM dealt with the health-focused problems and worked with the SW to address the other problems. The NCM then referred the participants to the TCM and a general practitioner according to the team-developed operational guidelines and referral protocols.

During the first Zoom meeting, the NCM performed an initial assessment, then taught the participants the skills required to perform self-care for health maintenance, including self-monitoring of vital signs, adhering to medication, and soliciting help if needed. Based on each participant’s problems, the NCM provided individualized health education covering recognition of the early signs and symptoms of exacerbation or deterioration of disease condition; the frequency, dosage, and duration of each health-promoting activity of therapeutic value; and the techniques required to perform these activities. The NCM helped the participants build their self-care confidence based on Bandura’s social cognitive theory [35] and engaged them to co-produce realistic, achievable goals. After the first Zoom meeting, the NCM and CWs conducted follow-up telephone calls and Zoom meetings to evaluate the progress of the participants and provide support when necessary. The NCM encouraged the participants to maintain ongoing self-care behavior, provided health advice, assessed their need for referral, and reviewed their health and social goals during telephone calls. The CWs supported the NCM in monitoring the progress of the participants in accordance with their contract goals, providing social support, and mobilizing community resources available in the district as appropriate with the help of the SW. Zoom meetings and telephone calls were used as the delivery channels, as previous studies have shown that such a combined approach is more effective than a single approach [20] (Additional file 1). Lastly, the NCM conducted a final Zoom meeting to conclude the intervention and perform a final assessment and health reinforcement in the third month.

As the health–social partnership was an important component of this program, Gittell’s relational coordination theory was used as a guide to achieve a better climate for teamwork among the NCM, CWs, and SW [31]. The intervention, developed using the theoretical guide, involved conducting regular interdisciplinary case conferences, ensuring adherence to the standardized protocol, and co-designing the care protocol, referral forms, and case records using the team approach. The roles and responsibilities of each member of the team specified in the protocol were established by consensus among the health and social team members. For example, the social workers provided home and meal delivery services, counseling, and financial support, while the TCM provided nutritional advice, and physical exercise and health counseling to the participants. Previous study has already confirmed the importance of these providers in promoting self-care of community-dwelling older adults [24]. Table 1 presents how the strategies were developed according to the conceptual guide discussed above.

**Table 1:** Effectiveness strategies in the intervention group

Theories	Content	Strategies
The Omaha system	Problem classification scheme	▪ Assess four domains, namely, environmental, psychosocial, physiological, and health-related behavior
	Intervention scheme	▪ Set contract goals and formulate an individual care plan with the participants ▪ Provide information about health-promoting and self-care activities
	Problem-rating scale for outcomes	▪ Evaluate knowledge, behavior, and status after implementing the intervention
Bandura’s social cognitive theory	Mastery experience	▪ Explore past successful experiences of handling health care issues ▪ Remind them of helpful strategies
	Vicarious experience	▪ Show pictures, newspaper clips, or videos of celebrities who have successfully adhered to self-care behavior
	Social and verbal persuasion	▪ Provide verbal encouragement
	Physiological and affective states	▪ Monitor and note the physiological status, i.e., vital signs, regularly in a booklet ▪ Encourage the participants to state their concerns about work
Gittell’s relational coordination theory	Routines	▪ Formulate a standardized protocol
	Information systems	▪ Create referral forms and records
	Meetings	▪ Conduct bimonthly case conferences (frequency can be adjusted)
	Boundary spanner	▪ The nurse case manager can provide strong leadership and help to integrate the work of others

#### Implementation part

This study adopted Durlak and DuPre’s implementation framework, which highlights that the successful implementation of a program is driven by five factors, namely, prevention delivery, prevention support, innovation characteristics, provider characteristics, and community factors [36].

The prevention delivery system addresses the capacity of a community center to successfully adopt a new program. The current program had received approval for further collaboration from the community center during the first meeting between the research team and the leaders and managers of the community center. Potential benefits to the center, such as increasing the center’s capacity to serve the needs of its members, improving the health knowledge of the staff, and enabling the members to live independently in the community, were discussed.

Apart from helping to build the center’s organizational capacity, the research team provided program orientation in the second meeting and continued to support the center throughout the study. This served as a factor contributing to the prevention support system (i.e., training and technical assistance). Topics of the meeting included an overview of the study rationale, timeline, roles and responsibilities of each member, recruitment process, and referral criteria. The research team provided continued telephone or one-on-one consultation support for the staff members for any difficulties they encountered during the study.

To reach the innovation level for a new program [36], monthly meetings with the providers, managers, and staff of the community center were held during the 3-month program to consolidate the educational contents and protocols and evaluate the feasibility of the program. In addition, the logistics of the program and the demographic background and preferences of the participants, providers, and staff and managers of the community center were integrated into the intervention and referral protocols. Modifications to the program were made to align with the center’s mission, preferences, and existing practices and community needs. The intervention and referral protocols were developed collaboratively by the research team and the service center staff to meet the needs of the health–social team, organizations, and communities.

According to Durlak and DuPre [36], providers who feel more confident in their ability and have the requisite skills to provide care for their clients are more likely to implement a program with higher levels of fidelity. To improve these provider characteristics, multiple training sessions on the implementation process, theoretical knowledge, practical skills, documentation processes, and the support system were provided for the NCM and center service providers (the administrative staff, SW, and CWs) before the commencement of the program.

To increase the likelihood of successfully delivering the program in the community, community factors including policy and funding must be considered. The Hong Kong government has instituted several policies to provide support and funding for non-governmental organizations to address the health and social needs of community-dwelling older adults [37]. The current HSPP may not only promote elderly healthcare services and provide support for aging but also provide guidance for the center to seek government support to sustain the program in the real-world setting. Table 2 highlights the implementation strategies for the program.

**Table 2:** Implementation strategies in the intervention group

Categories	Variables	Strategies
Prevention delivery system	Leadership/managerial support	The impact, pros, and cons of the Health–Social Partnership Program were discussed during a meeting with the managers of the community center. They perceived the program highly favorably, with potential to be implemented long term in the center.
	Communication/coordination with other agencies	The research team set up meetings with agencies, including the Senior Citizen Home Safety Association, and general practitioners to discuss the possibilities of integrating their services into the program.
Prevention support system	Training	Before the commencement of the program, the health–social team received training, through a formal presentation, on the implementation process, theoretical knowledge, practical skills, referral criteria, and documentation processes.
	Technical assistance	The research team organized a monthly cross-service team meeting via videoconferencing to discuss the progress and challenges and offer support. Training was provided for new staff members in case of staff attrition.
Innovation characteristics	Adaptability	Multiple meetings were conducted with the manager and staff of the community center to seek their input to modify the program to fit their preferences, organizational practices, and community needs.
	Compatibility	The research team and the community center staff collaboratively developed the intervention and referral protocols before the commencement of the program.
Provider characteristics	Self-efficacy/skill proficiency	Training, encouragement, and ongoing support were provided for the health–social team. The team members were required to show return demonstrations to assess their competencies.
Community factors	Policy	The Hong Kong government acknowledges the importance of primary healthcare services and provides support and funding for non-governmental organizations to address the health and social needs of citizens.
	Funding	The community center plans to apply for government funding to support the maintenance of the program after the pilot study.

### Control group

Both the intervention and control groups received usual community services. In the community center, regular health talks and basic health checks such as measuring blood pressure, blood glucose, and body fat percentage were accessible to all residents. Participation was voluntary. The participants in the control group received a monthly social control call from a trained research assistant to rule out possible social effects of the intervention.

### Outcome measures

Below is a description of the measures reflecting the reach, effectiveness, adoption, implementation, and maintenance (RE-AIM) dimensions [38]. The effectiveness dimension examined the outcome measures of both the intervention and control groups. All other dimensions were only relevant to the intervention group.

Indicators of reach included the recruitment rate and participant characteristics. To calculate the recruitment rate, the total number of recruited participants was divided by the total number of eligible participants from the member list of the community center. The demographic data of the participants in this study were compared with data from the Census and Statistics department to identify the representativeness of our sample. The effectiveness of the program was measured using self-efficacy as the primary outcome and quality of life and health service utilization as the secondary outcomes. Self-efficacy was measured using the Chinese version of the General Self-efficacy Scale. The scale was previously validated in the Chinese population and has a reliability alpha coefficient of 0.89 [39]. Quality of life was measured using the 12-item Short Form Health Survey version 2 – Chinese (HK) version (SF-12v2HK) [40]. The scale has been used in numerous studies, and its reliability has been confirmed in the local population [24]. The outcomes of health service utilization included the numbers of unscheduled general out-patient department visits, general practitioner visits, emergency department visits, and hospital admissions and the total number of health service uses. The data were self-reported by the participants and confirmed against the medical records and attendance certificates, with good reliability [24]. The facilitating factors of and barriers to program adoption were explored through semi-structured group interviews of the center staff members, managers, providers, and participants. The extent to which the intervention was implemented as intended was analyzed using a performance checklist by a research team member, who was not involved in implementation, during random quality assurance Zoom meetings with the NCM. The effects of the program on the primary and secondary outcomes 3 months after the completion of the program were measured as indicators of program maintenance.

### Data collection

Data were collected at three time intervals: pre-intervention (T1), immediately post-intervention (T2), and 3 months post-intervention (T3). The data were collected by a trained student assistant who was blinded to group allocation and not involved in intervention delivery in the intervention or the control group. The study flow chart is provided in Additional file 1.

To examine the facilitators of and barriers to program adoption, semi-structured interviews were conducted by the research team members at T2. A one-time, in-depth focus group interview for each group—(i) center managers and staff, (ii) service providers (i.e., the SWs and NCM), and (iii) eight (20%) of the intervention group participants—allowed the research team to understand the group dynamics by exploring the opinions of the different groups. A study [41] indicated eight as the optimal number of people in a focus group to ensure the group is small enough to hear the voices of all participants and large enough to collect a variety of perspectives. The semi-structured interview guide was developed based on the Social Ecological Model (SEM) [29]. This model provides a framework to understand multilevel factors related to the acceptability, perceived facilitating factors of and barriers to program adoption in the community centers. Examples of questions included “what are some personal factors that make it easier or difficult to receive the program service in the center?” and “how community center can help facilitate/hinder the implementation of the program to the older adults?”

### Data analysis

A generalized estimating equation was used to calculate the changes in or differences between the intervention and control groups (between-group effects), within-group (time) effects, and interaction effects (group × time). A linear link function was used to examine the self-efficacy and quality of life of the participants. The Poisson link function was used to examine the mean differences in health service utilization. The working correlation was first-order autoregressive. Intention-to-treat analysis was performed as the primary analysis.

The qualitative data gathered in the three semi-structured interviews were analyzed using thematic analysis with a deductive approach [42]. All focus group interviews were audio-recorded, and the data were transcribed by the research team members. The research team members independently examined the raw text and identified relevant themes. The team discussed and constructed a framework for analysis with codes and categories clearly defined, which helped to summarize the data to answer the research questions [43]. Following these, similar codes were summarized to generate sub-themes. The research questions for the qualitative phase were used as heuristic guides to generate higher order themes. An audit trail was kept to ensure the consistency of coding and interpretation, and all discrepancies were resolved by consensus.

### Trustworthiness and methodological rigour

The framework of Lincoln and Guba [44] was employed to attain trustworthiness in the qualitative part of the study. This trustworthiness framework seeks to attain rigour using four constructs: credibility, transferability, dependability, and confirmability. Credibility refers to the fit between participants’ descriptions and their representations by the research team. To attain this, the interviewer employed probes, prompts, and an iterative mode of questioning throughout the interview process. Additionally, the interviewer demonstrated competency in qualitative interviewing prior to participating in the study to ensure that required data are obtained. Repeat interviews, where possible were conducted to clarify unclear issues from the preceding interview. Native Chinese speakers who are fluent in English also completed the interviews. During the analytical process, an audit trail was maintained to trach all methodological decisions. Transferability refers to how the study findings can fit into other contexts beyond the current study setting. To attain this, the authors have provided a thick description of the study processes and emerging findings to enable readers to judge the overall application of the findings to their context. Dependability concerns itself with the researcher’s responsibility to substantiate that every part of the study is methodical and transparent. To attain this, details regarding the entire research process were documented and highlighted in this manuscript. Confirmability refers to the accuracy of data and soundness of decisions throughout the study. To achieve this, it was ensured that only participants who met the eligibility criteria participated in the interviews. Discussion of the study findings with participants enabled to research team to confirm the interpretations thereof. Also, the involvement of native speakers who are also fluent in English and ongoing consultation with the wider research team ensured that the study was conducted rigorously.

For the quantitative aspect of the study, rigour was ensured by following standard procedures regarding participant recruitment, delivery of the intervention, and data collection through blinding and randomization [45]. Also, the data analytical approach employed was commensurate to the research objectives.

## Results

### Demographic characteristics of the participants

Ninety-two older adults participated in this study. As shown in Table 3, the mean age of the participants was 75.9 years, 84.8% of the participants were female, and 44.6 and 45.7% of the participants were married and widowed, respectively. The sociodemographic data of the intervention and control groups were comparable.

**Table 3:** Sociodemographic characteristics of the study participants

Variable	Control (*n* = 46)	Intervention (*n* = 46)	All (*N* = 92)
	N (%)	N (%)	N (%)
Age, years (mean, SD)	78.2 (8.1)	73.7 (6.9)	75.9 (7.8)
Sex			
Female	39 (84.8)	39 (84.8)	78 (84.8)
Male	7 (15.2)	7 (15.2)	14 (15.2)
Marital status			
Married	18 (39.1)	23 (50)	41 (44.6)
Widowed	23 (50)	19 (41.3)	42 (45.7)
Divorced	4 (8.7)	2 (4.3)	6 (6.5)
Single	1 (2.2)	2 (4.3)	3 (3.3)
Education			
No schooling	15 (32.6)	4 (8.7)	19 (20.7)
Primary	19 (41.3)	23 (50)	42 (45.7)
Secondary	11 (23.9)	19 (41.3)	30 (32.6)
University	1 (2.2)	0 (0)	1 (1.1)
Employment			
Employed	0 (0)	0 (0)	0 (0)
Retired	46 (100)	46 (100)	92 (100)
Housing type			
Flat	46 (100)	46 (100)	92 (100)
Subdivided flat	0 (0)	0 (0)	0 (0)
Cage Home	0 (0)	0 (0)	0 (0)
Household			
Living alone	13 (28.3)	10 (21.7)	23 (25)
Living with elderly spouse	9 (19.6)	10 (21.7)	19 (20.7)
Living with family	24 (52.2)	26 (56.5)	50 (54.3)
Perceived economic status			
More than enough	14 (30.4)	9 (19.6)	23 (25)
Just enough	31 (67.4)	32 (69.6)	63 (68.5)
Not enough	1 (2.2)	4 (8.7)	5 (5.4)
Inadequate	0 (0)	1 (2.2)	1 (1.1)
Source of income			
Provided by family	34 (73.9)	23 (50)	57 (62)
Self-savings	10 (21.7)	11 (23.7)	21 (22.8)
Retirement pension	4 (8.7)	3 (6.5)	7 (7.6)
Comprehensive Social Security Assistance	0 (0)	2 (4.3)	2 (2.2)
Old Age Living Allowance	34 (73.9)	29 (63)	63 (68.5)
Source of care			
Self	37 (80.4)	37 (80.4)	74 (80.4)
Spouse	11 (23.9)	9 (19.6)	20 (21.7)
Siblings	0 (0)	4 (8.7)	4 (4.3)
Children	23 (50)	23 (50)	46 (50)
Children in law	4 (8.7)	5 (10.9)	9 (9.8)
Volunteers	3 (6.5)	3 (6.5)	6 (6.5)
Domestic helpers	8 (17.4)	1 (2.2)	9 (9.8)
Frequency of care			
Always	33 (71.7)	21 (45.7)	54 (58.7)
Sometimes	7 (15.2)	10 (21.7)	17 (18.5)
Only at night	2 (4.3)	5 (10.9)	7 (7.6)
No help from others	4 (8.7)	10 (21.7)	14 (15.2)

### Reach

**Fig. 1:**
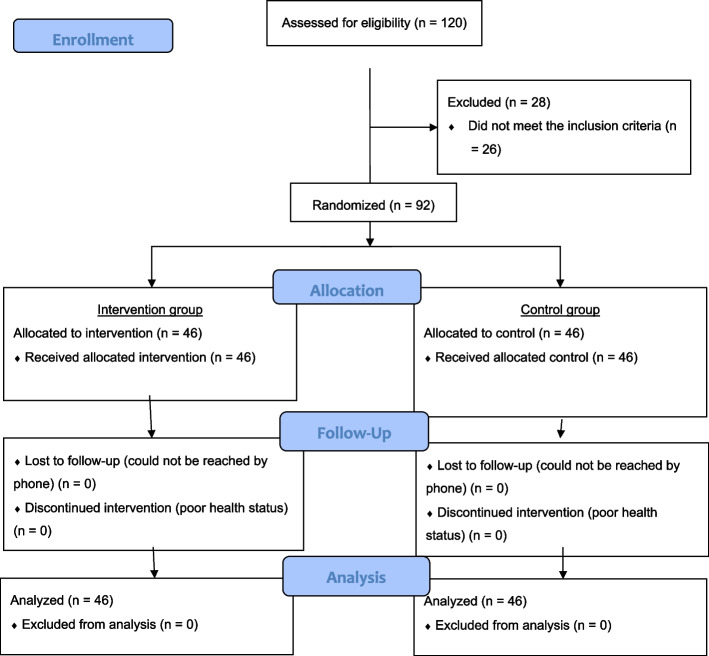
CONSORT table

The CONSORT table (Fig. 1) shows that the number of eligible participants in the community center was 120. As eventually 92 participants joined and completed the program, the recruitment rate was 76.7%. In addition, the demographic characteristics were not significantly different between the participants and the non-enrolled eligible participants. Table 4 displays the clinical characteristics of the participants. The most common conditions affecting the participants were hypertension (56.5%), diabetes mellitus (17.4%), and high cholesterol (15.2%); these were also the three most prevalent chronic conditions reported in the latest local Census survey (2021) [46]. In addition, 36.23% of the participants reported that they had consulted a doctor in the past 30 days and 88% of the participants usually visited Western medicine practitioners; these percentages are similar to the ratios reported in the 2021 Census survey. This program successfully reached the target population, i.e., older adults that live independently in the community.

**Table 4:** Comparison of the health status, doctor visits, and hospitalization records with the 2021 Census report

Variable	Control (*n* = 46)	Intervention (*n* = 46)	All (*N* = 92)	Census and Statistics Department (Dec, 2021)
	N (%)	N (%)	N (%)	%
*Chronic conditions				
Hypertension	24 (52.2)	28 (60.9)	52 (56.5)	67.0%
Diabetes mellitus	9 (19.6)	7 (15.2)	16 (17.4)	29.9%
High cholesterol	8 (17.4)	6 (13.0)	14 (15.2)	34.3%
Heart diseases	5 (10.9)	4 (8.7)	9 (9.8)	11%
Cancer	3 (6.5)	2 (4.3)	5 (5.4)	4.7%
Stroke	1 (2.2)	1 (2.2)	2 (2.2)	4.1%
Respiratory/asthma	1 (2.2)	1 (2.2)	2 (2.2)	2.0%
Others (pain, genital conditions, arthritis, depression, fracture, cataract, etc.)	27 (58.7)	25 (54.3)	52 (56.5)	52.4%
^#^Usually visited doctor				
Practitioners of Western medicine only	38 (82.6)	43 (93.5)	81 (88.0)	86.6%
Practitioners of Chinese medicine only	8 (17.4)	6 (13.0)	14 (15.2)	5.9%
Practitioners of both Western and Chinese medicine	0 (0)	3 (6.5)	3 (3.3)	7.4%
*Consulted a doctor in the past 30 days	14.0 (30.4)	18.7(40.6)	16.6 (36.2)	31.3%
^a^Total number of consultations	86	127	223	1681
Visited clinics/centers under Hospital Authority or Department of Health	21 (24.4)	24 (18.9)	45 (20.2)	40.8
Visited private practitioners	58 (67.4)	93 (73.2)	161 (72.2)	56.6
Visited accident and emergency department	7 (8.1)	10 (7.9)	17 (7.6)	1.6
Hospital admissions in the past 12 months	3 (6.5)	9 (19.6)	12 (13.0)	13.1%

^a^Sum of three time-points

^*^Compared with the ≥65-year age group

^#^Compared with all age groups

### Effectiveness

#### Self-efficacy

**Fig. 2:**
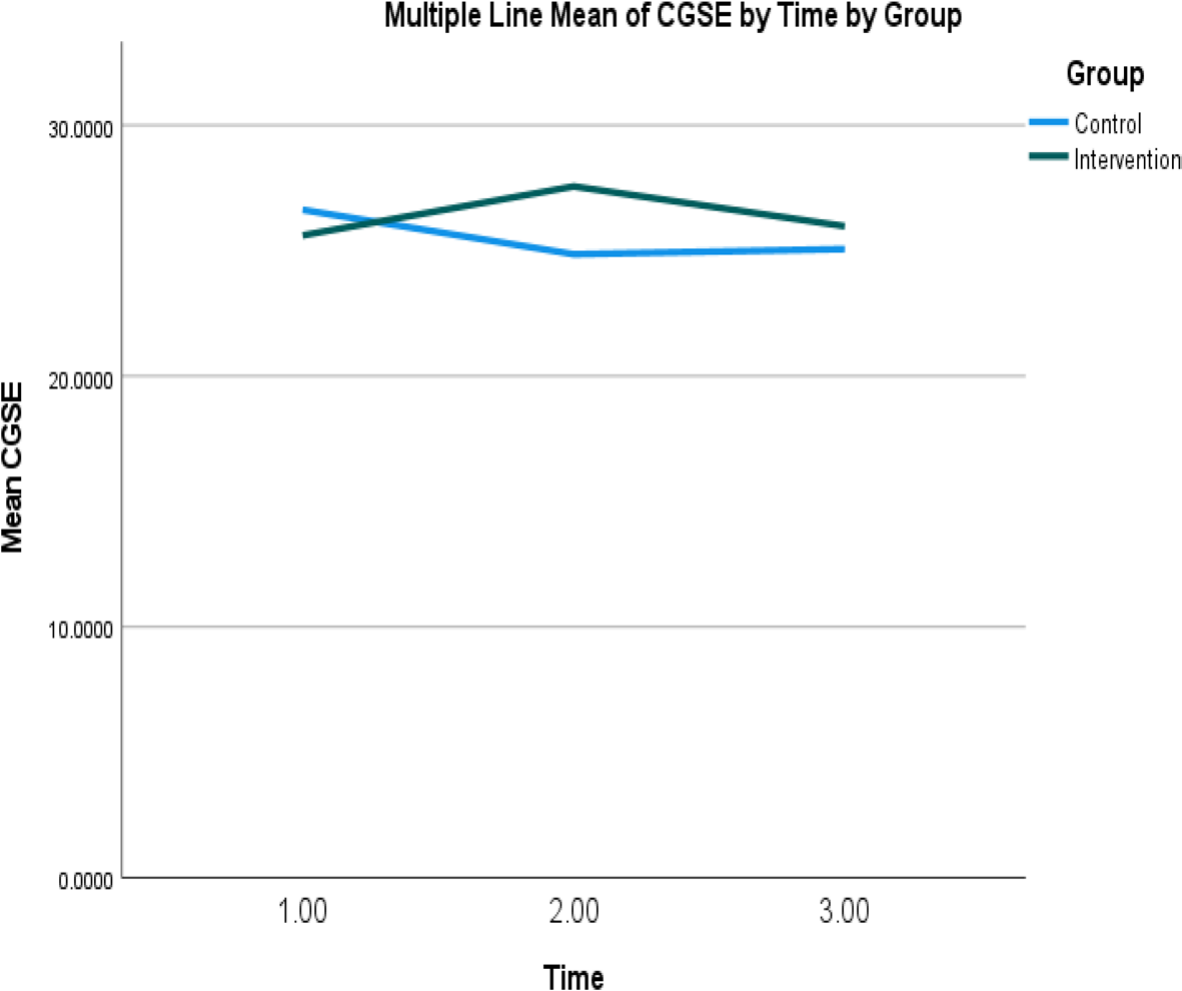
Mean self-efficacy scores of intervention and control groups over time. Note: Higher scores indicate higher levels of self-efficacy level

Table 4 shows the mean self-efficacy scores for the intervention and control groups at T1 and T2. A significant interaction effect was observed between the two groups at T1 and T2 (Wald χ^2^ = 12.28, *p* ≤ .001); particularly, the mean self-efficacy scores increased from T1 to T2 in the intervention group but decreased from T1 to T2 in the control group (Table 5 and Fig. 2).

**Table 5:** The mean and standard error of the effectiveness outcomes for the intervention and control groups at three time-points

Outcomes	Groups		Mean	Standard error	95% Wald confidence interval	
					Lower	Upper
Self-efficacy	Control group	T3	25.07	0.67	23.76	26.37
		T2	24.86	0.88	23.13	26.59
		T1	26.63	0.87	24.92	28.34
	Intervention group	T3	25.98	0.68	24.65	27.31
		T2	27.56	0.77	26.05	29.07
		T1	25.61	0.77	24.10	27.13
Quality of life – Physical component scores	Control group	T3	43.90	1.39	41.18	46.62
		T2	42.59	1.38	39.89	45.29
		T1	41.78	1.41	39.01	44.54
	Intervention group	T3	45.10	1.37	42.43	47.78
		T2	45.95	1.09	43.82	48.08
		T1	42.68	1.21	40.30	45.05
Quality of life – Mental component scores	Control group	T3	50.37	1.21	48.01	52.74
		T2	51.28	1.23	48.87	53.69
		T1	51.54	1.24	49.11	53.96
	Intervention group	T3	47.27	1.33	44.67	49.87
		T2	48.94	1.30	46.38	51.49
		T1	48.47	1.22	46.08	50.87
Total unplanned health service uses	Control group	T3	0.43	0.13	0.23	0.79
		T2	0.65	0.26	0.30	1.41
		T1	0.79	0.19	0.49	1.29
	Intervention group	T3	0.67	0.16	0.42	1.07
		T2	1.06	0.28	0.63	1.79
		T1	1.57	0.39	0.96	2.56
Unplanned General Out-Patient Department visits	Control group	T3	0.09	0.05	0.03	0.28
		T2	0.12	0.06	0.04	0.32
		T1	0.28	0.10	0.14	0.56
	Intervention group	T3	0.15	0.09	0.05	0.50
		T2	0.17	0.08	0.07	0.43
		T1	0.18	0.14	0.04	0.85
Unplanned general practitioner visits	Control group	T3	0.30	0.12	0.14	0.67
		T2	0.50	0.25	0.19	1.34
		T1	0.41	0.13	0.22	0.76
	Intervention group	T3	0.46	0.13	0.26	0.81
		T2	0.69	0.27	0.32	1.49
		T1	1.12	0.30	0.67	1.88
Unplanned emergency department visits	Control group	T3	0.02	0.02	0.00	0.15
		T2	0.06	0.03	0.02	0.18
		T1	0.07	0.04	0.03	0.19
	Intervention group	T3	0.04	0.03	0.01	0.17
		T2	0.10	0.04	0.05	0.24
		T1	0.10	0.05	0.04	0.28
Unplanned hospital admissions	Control group	T3	0.02	0.02	0.00	0.15
		T2	0.02	0.02	0.00	0.14
		T1	0.04	0.03	0.01	0.14
	Intervention group	T3	0.02	0.02	0.00	0.15
		T2	0.10	0.05	0.04	0.28
		T1	0.16	0.07	0.07	0.36

T1: pre-intervention; T2: immediately post-intervention; T3: 3 months post-intervention

### Quality of life

**Fig. 3:**
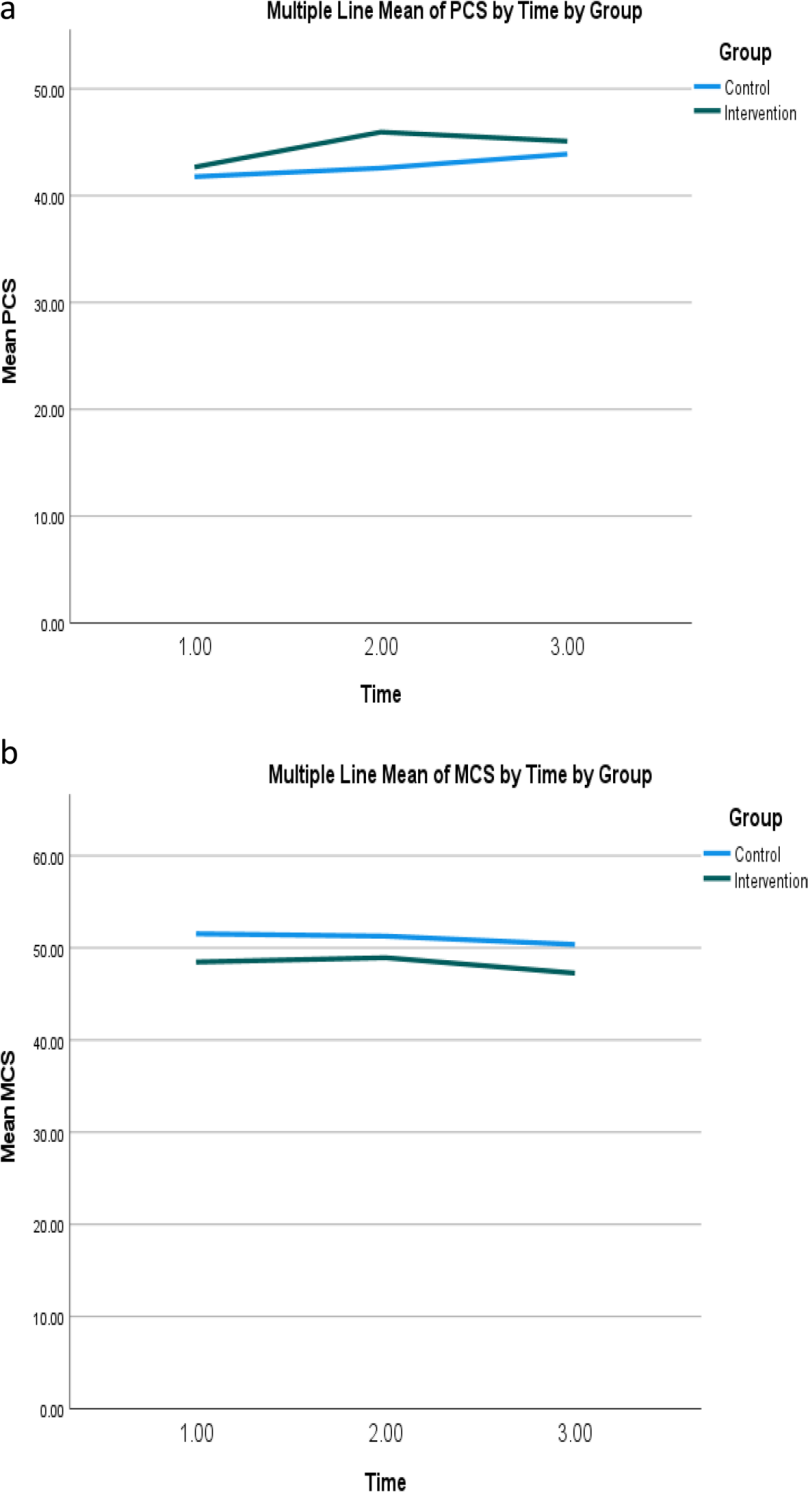
**a** Mean physical component summary scores of quality of life of intervention and control groups over time. Note: Higher scores indicate higher levels of physical component of quality of life. **b** Mean mental component summary scores of quality of life of intervention and control groups over time. Note: Higher scores indicate higher levels of mental component of quality of life

Although the physical component scores (PCS) of the intervention group improved from T1 to T2, no statistically significant within-group effect was observed (Fig. 3a). Further examination of the mean and *p* values also confirmed that there were no between-group, within-group, and interaction effects in the PCS and mental component scores between the two groups at T1 and T2 (Fig. 3b).

### Health service utilization

The outcomes of health service utilization included the numbers of unscheduled general out-patient department visits, general practitioner visits, emergency department visits, and hospital admissions, and the total number of health service uses. Table 5 demonstrates that in both the intervention and control groups, the use of health services decreased from T1 to T2, except for the number of unplanned general practitioner visits, which decreased sharply in the intervention group but increased in the control group from T1 to T2. However, no significant between-group, within-group, and interaction effects were observed in any of the health service utilization outcomes (Table 6).

**Table 6:**
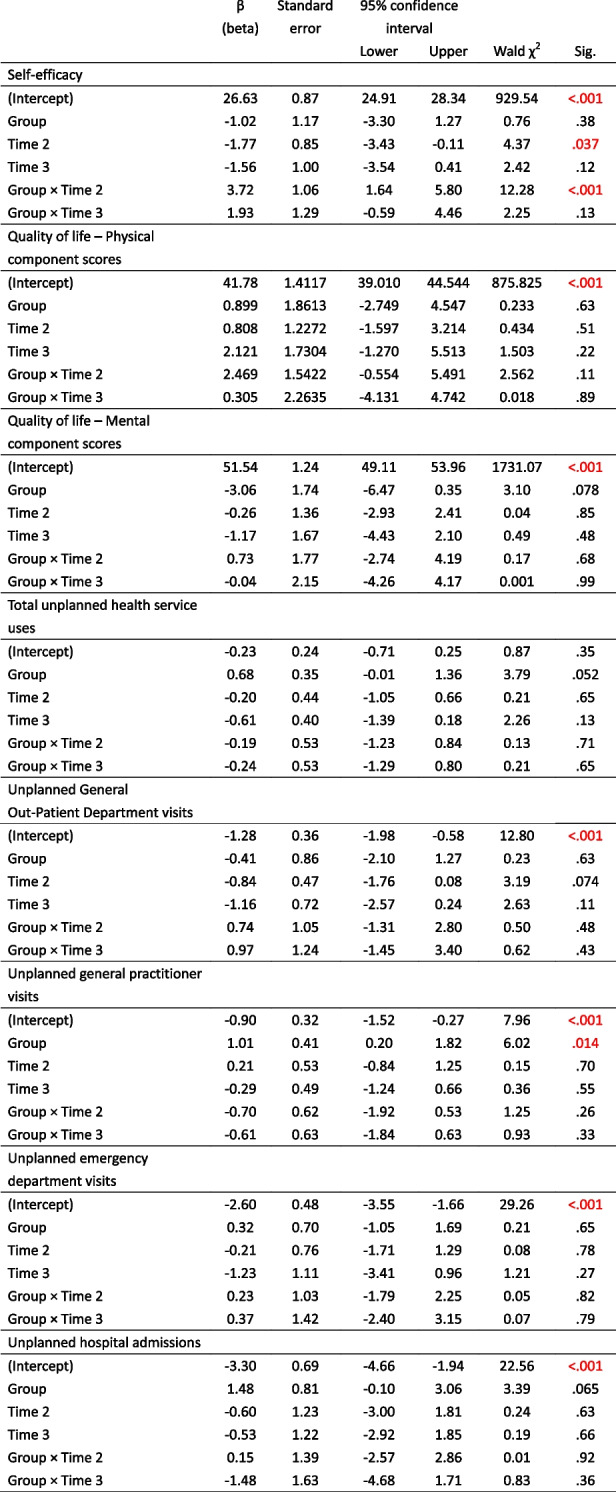
Parameter estimates for effectiveness outcomes

Time 2: immediately post-intervention; Time 3: 3 months post-intervention

Numbers in red color indicate statistically significant results

### Adoption

Three semi-structured interviews were conducted, one each with a center staff member a center manager, two providers, and eight participants, to discuss the facilitating factors of and barriers to program adoption. The themes and sub-themes are presented in Table 7.

**Table 7:** Themes and sub-themes of Adoption

Themes	Sub-themes
Barrier to program adoption in the community centre	1. Impact of COVID-19 pandemic 2. Existing service-related issues
Facilitators of program adoption in the community centre	1. Collaboration with gatekeepers 2. Promoting the program among staff 3. Consensus building and communication

#### Theme 1: barriers to program adoption in the community Centre

##### Impact of COVID-19 pandemic

Prior to the emergence of the COVID-19 pandemic, older adults relied heavily on advertisements issued by the community centres to be able to identify programs they could participate in. The pandemic and the need to minimize risk however led to the closure of the community centers indicating that their source of information regarding upcoming programs was cut-off. Participants highlighted that the center closure as a barrier to program adoption as they could not receive updates regarding the program which subsequently led to their non-participation:

*“We usually rely on promotional posters or notices by the community staff regarding any new programs that will be organized by the community center. The closure of the community center during the pandemic made it difficult for us to get updated information about new programs.” – [Participant].*

The effect of the pandemic affecting program adoption was reiterated by the center staff members and the manager. They mentioned that the pandemic led to significant changes in operation and service delivery, including re-deployment of staff which increased existing workload. Overall, these changes affected the planning and delivery of the new program:

*“We had a few meetings before the program launched. However, due to the pandemic, we have had to change the recruitment procedure, reschedule the duties of our staff, and revise the logistics of the program. All of this has increased our workload as we also need to make similar changes to other programs.” – [Community center staff manager].*

##### Existing service-related issues

Further to the above, the center staff manager highlighted key issues which served as barriers to implementing new programs and continuing with existing ones. These issues included limited staff strength to run a new program in addition to existing ones, availability of trained staff to deliver the program, limited government support in increasing the staff strength, and financial constraints. Put together, these issues adversely impacted on sustaining existing programs and successfully implementing new programs. Additionally, from the providers’ perspective, it was critical to have healthcare professionals specifically trained to deliver the program; a lack of which can adversely impact the planning and implementation of the program:

*“Manpower is the biggest issue for the center. While the research team has provided us with a nurse and research assistants, we still need additional staff to run a new program. Even for our regular programs, we need financial support from the government to hire a registered nurse and clerical staff.” – [Community center staff manager].*

*“This program requires an experienced and skilled nurse to monitor the health conditions of the participants, provide optimal and individualized interventions, and handle emergency situations. It will be more difficult to meet the standard of the program if the nurse is not specialized in community or elderly care. A nonspecialized nurse would need more guidance and training.” – [Provider].*

#### Theme 2: facilitators of program adoption in the community Centre

##### Collaboration with gatekeepers

Forging collaboration with the healthcare professionals at the community served as a facilitator for the adoption of the program among participants. Existing relationship and trust between community center staff and the older adults which may have existed for a while can potentially lead to participants accepting any recommendation from the staff. In this way, the community center staff will serve as gatekeepers and by including them in the study, participants are assured of good outcomes:

*“You said you are a university staff member. How can I trust you? There are so many swindlers in the world. However, if a community center staff member called me and recommended the program to me, I would immediately join the program without hesitation.” – [Participant].*

##### Promoting the program among staff

The participating manager re-echoed a need to reach out to all staff members and promote the program among them. In this way, staff members are kept abreast with firsthand information about the study as well as offering an opportunity to respond to other issues they may have so they can in turn be able to participate in the program:

*“Instead of us calling the members, it would be better if the research team could organize a briefing session in the community center. In the briefing session, the research team could talk about the aims, benefits, and requirements of the program. The research team members are the ideal people to answer questions from our members regarding the details of the program.” – [Community center staff manager].*

##### Consensus building and communication

Staff manager participants highlighted building consensus and communication as key ingredients to facilitate the adoption and implementation of a new program. Participants reiterated that this involved good-faith efforts to attain unanimous agreement between the research and service delivery teams to facilitate smooth implementation of the program. Participants recounted and reflected on previous experiences with the research team in a positive light and felt this may help to drive the collaboration forward to support the adoption of the program:

*“There were a lot of things to discuss before we could adopt the program here. Whether the mission and vision of the program are consistent with ours, the logistics and manpower issues, the equipment and instruments needed… The research–service team members have to reach a consensus before the program can be launched smoothly. In fact, we think that our experience of working with the research team was good because we had several fruitful discussions beforehand.” – [Community center staff manager].*

Regarding communication, the providers’ comments echoed the opinion of the community center staff manager, although the providers’ main focus was the interaction among the health–social partnership team members. The providers noted a need for constant communication across the service delivery team before, during, and after the implementation of the program:

*“Regular communication among the health–social partnership team members is crucial before the commencement of the program, as well as during and after the program. We learned to clearly understand each other’s working styles, roles, and responsibilities. When any participant’s condition changed, we could revise our action plans and protocols during our group meetings. The revised protocols are stored in the community center for reference.” – [Provider].*

### Implementation

A research team member, who did not participate in the intervention procedure, joined the Zoom meetings with the NCM and CWs at least twice weekly to evaluate the intervention fidelity using a performance checklist (Additional file 2). The completion rate of each of the 11 and 5 items in the performance checklists for the NCM and CWs, respectively, was high. The NCM received the highest rating of 100% in “Assess the participant holistically using the Omaha system” and “Encourage the participant to maintain ongoing self-care behavior using Bandura’s social theory,” whereas the CWs received the highest rating of 100% in “Provide social support in the Zoom meeting and follow-up telephone calls.” This indicated that the NCM and CWs adhered to the working protocols and delivered the interventions as intended.

### Maintenance

**Fig. 4:**
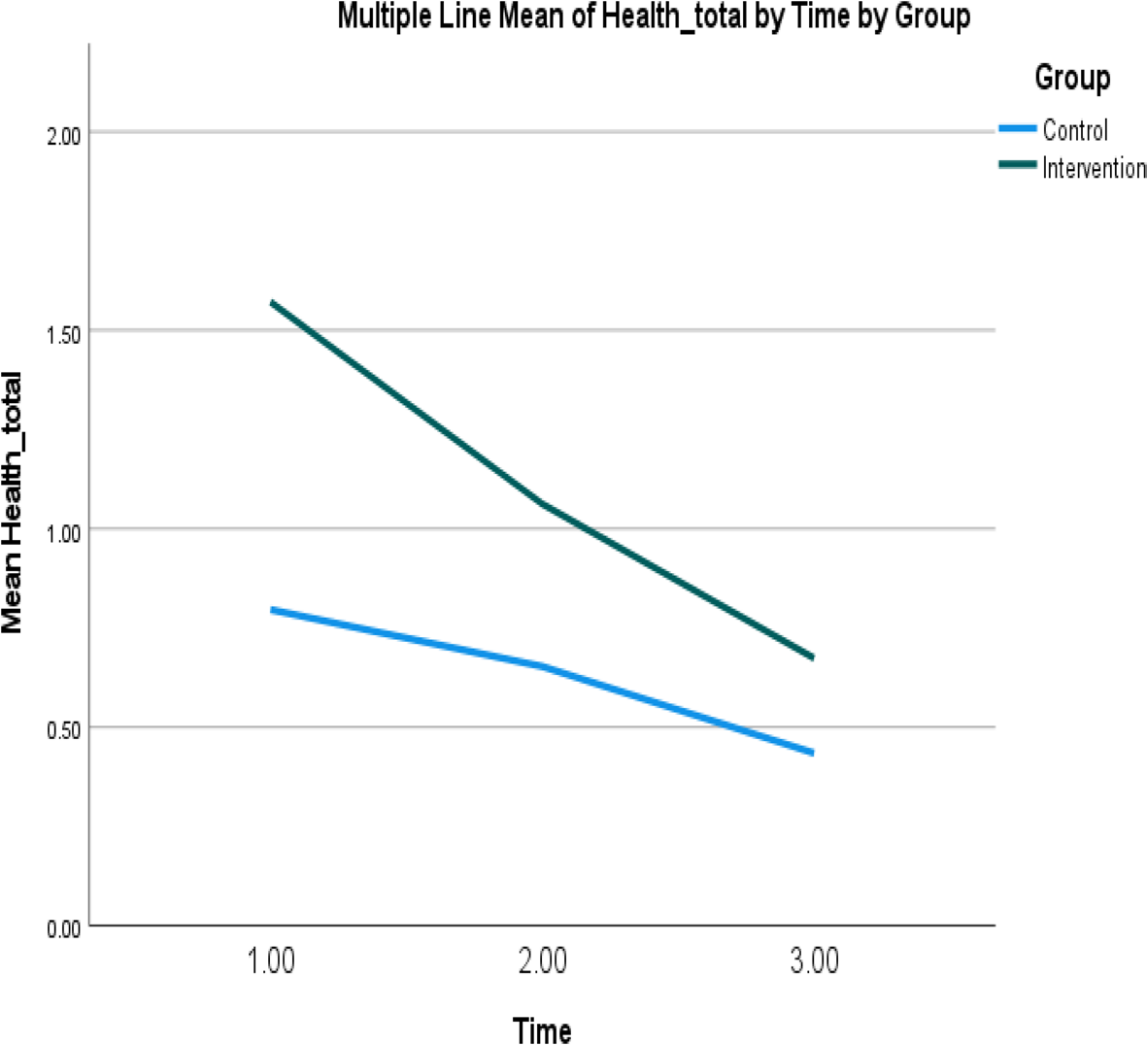
Mean of total health service utilization of intervention and control groups over time. Note: Higher mean scores indicate higher total health service utilizations

**Fig. 5:**
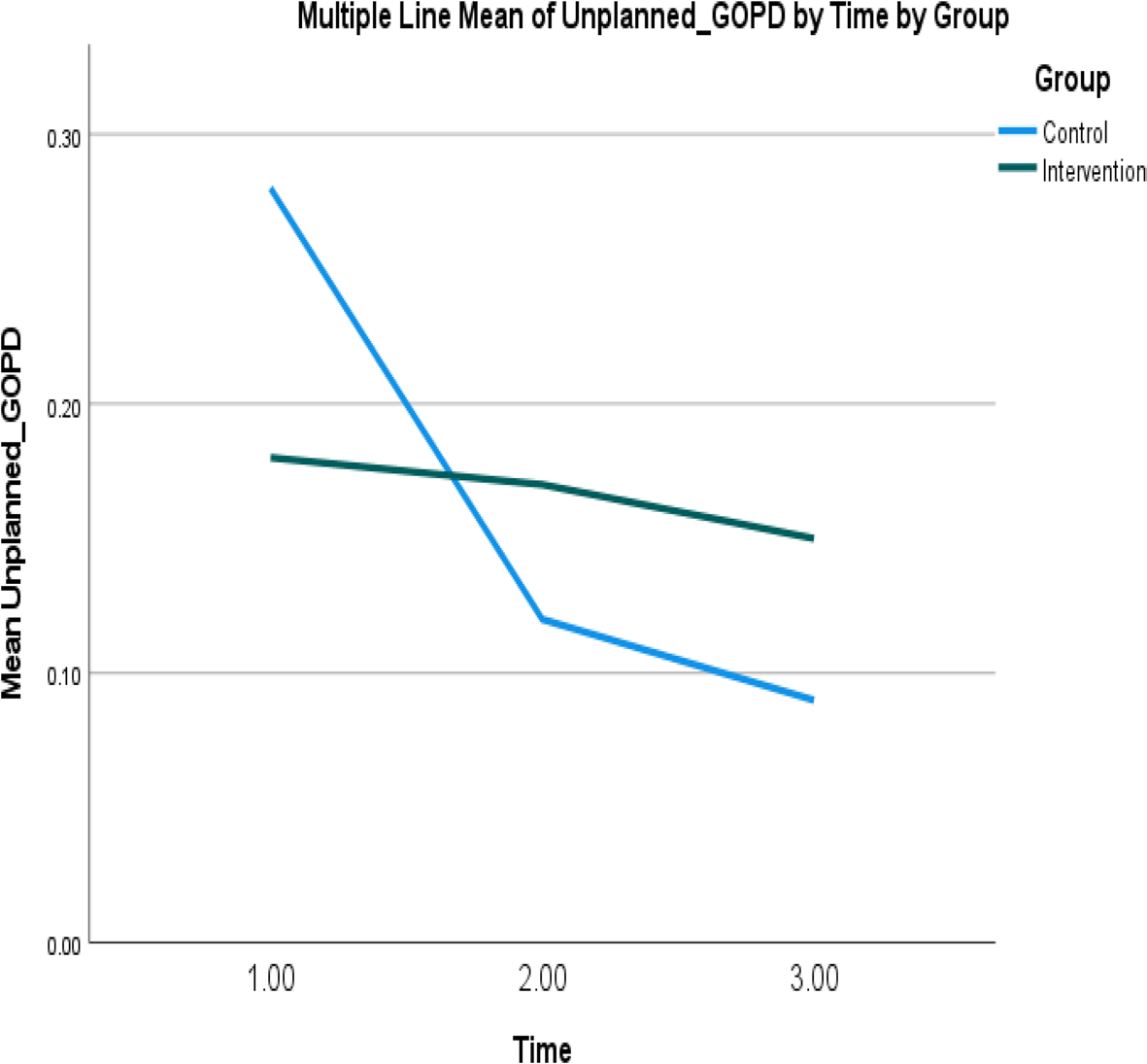
Mean of unplanned GOPD admissions of intervention and control groups over time. Note: Higher mean scores indicate higher unplanned GOPD admissions

**Fig. 6:**
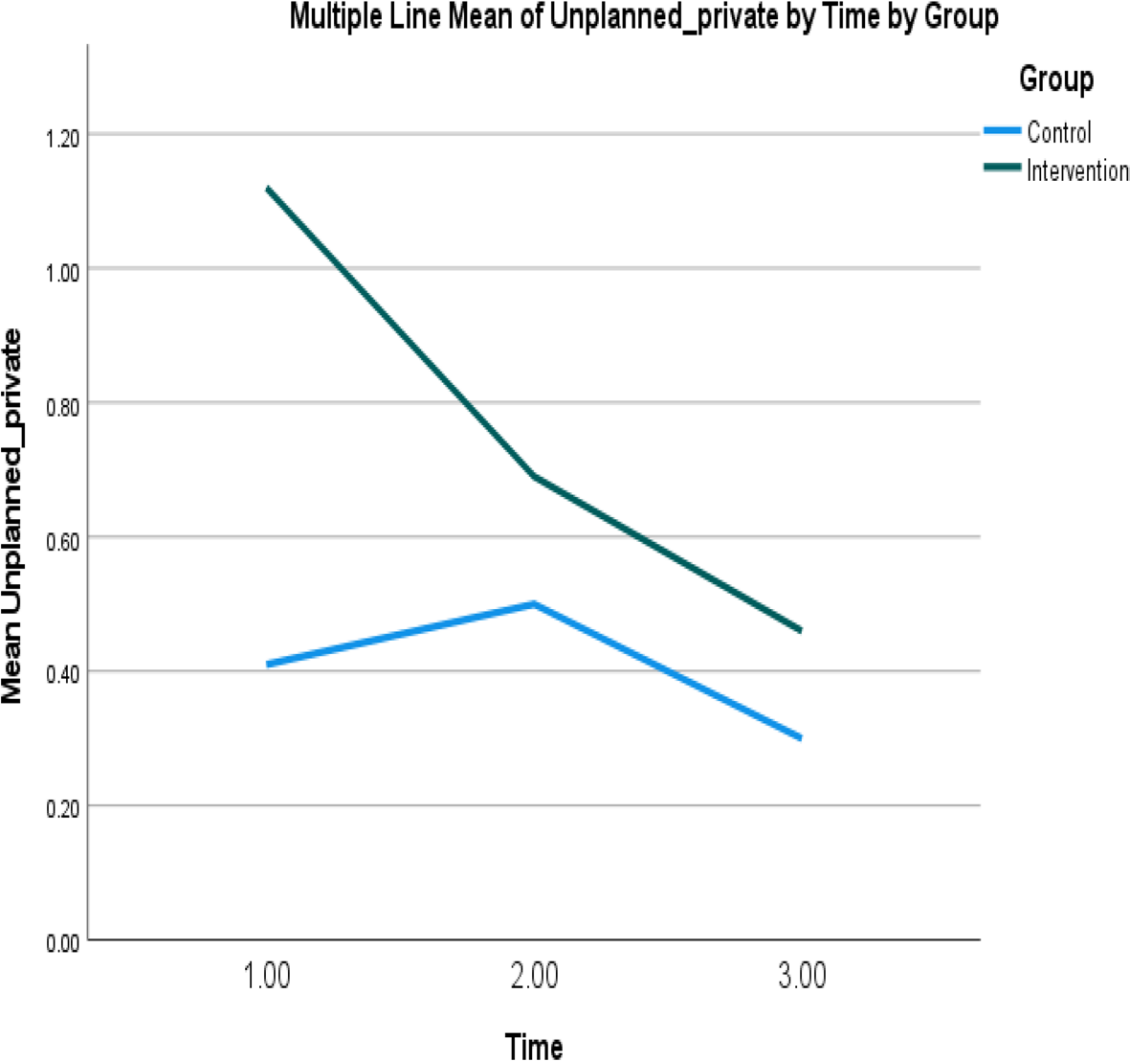
Mean of unplanned GP visits of intervention and control groups over time. Note: Higher mean scores indicate higher unplanned GP visits

**Fig. 7:**
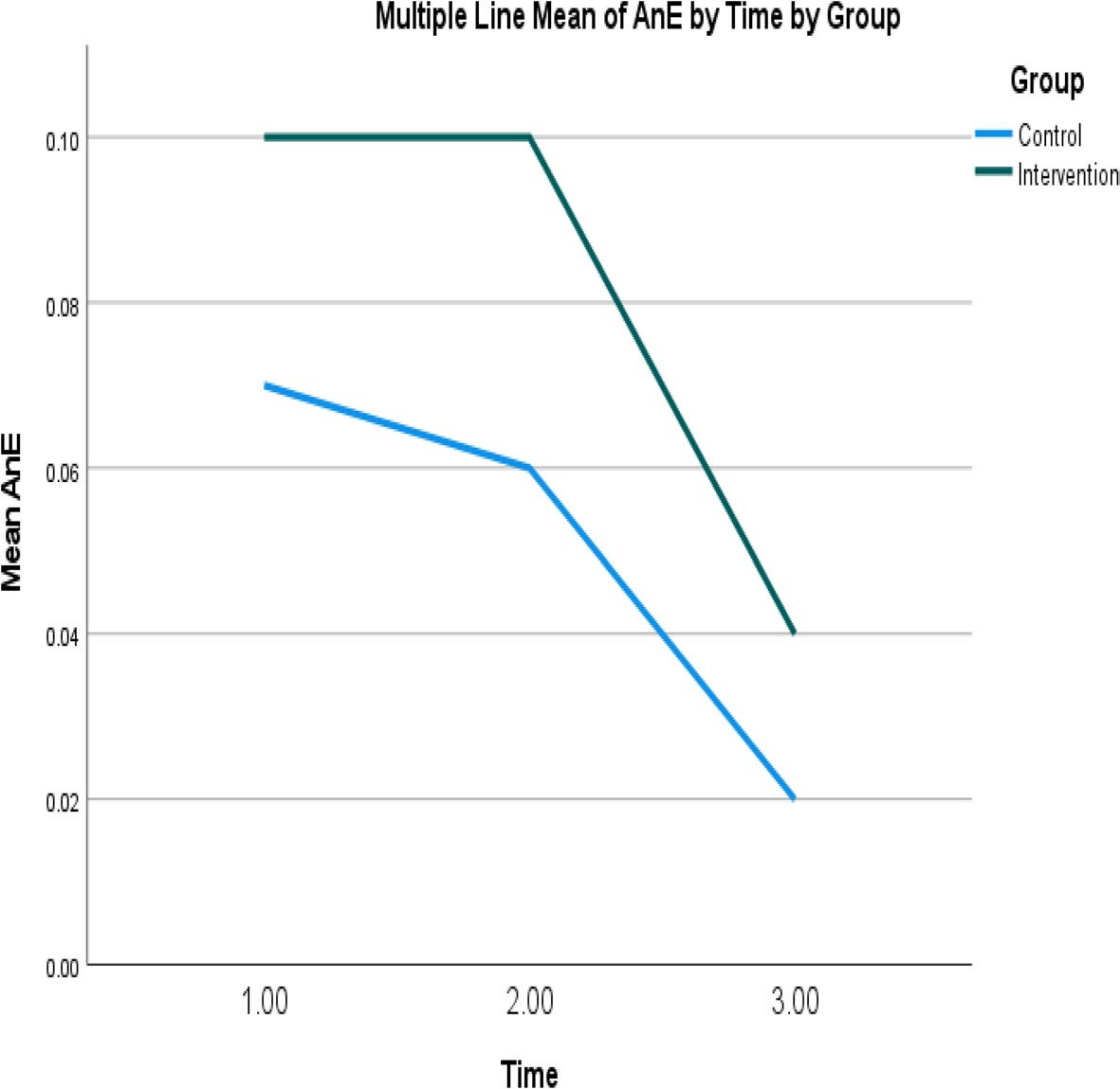
Mean of unplanned emergency department visits of intervention and control groups over time. Note: Higher mean scores indicate higher unplanned emergency department visits

**Fig. 8:**
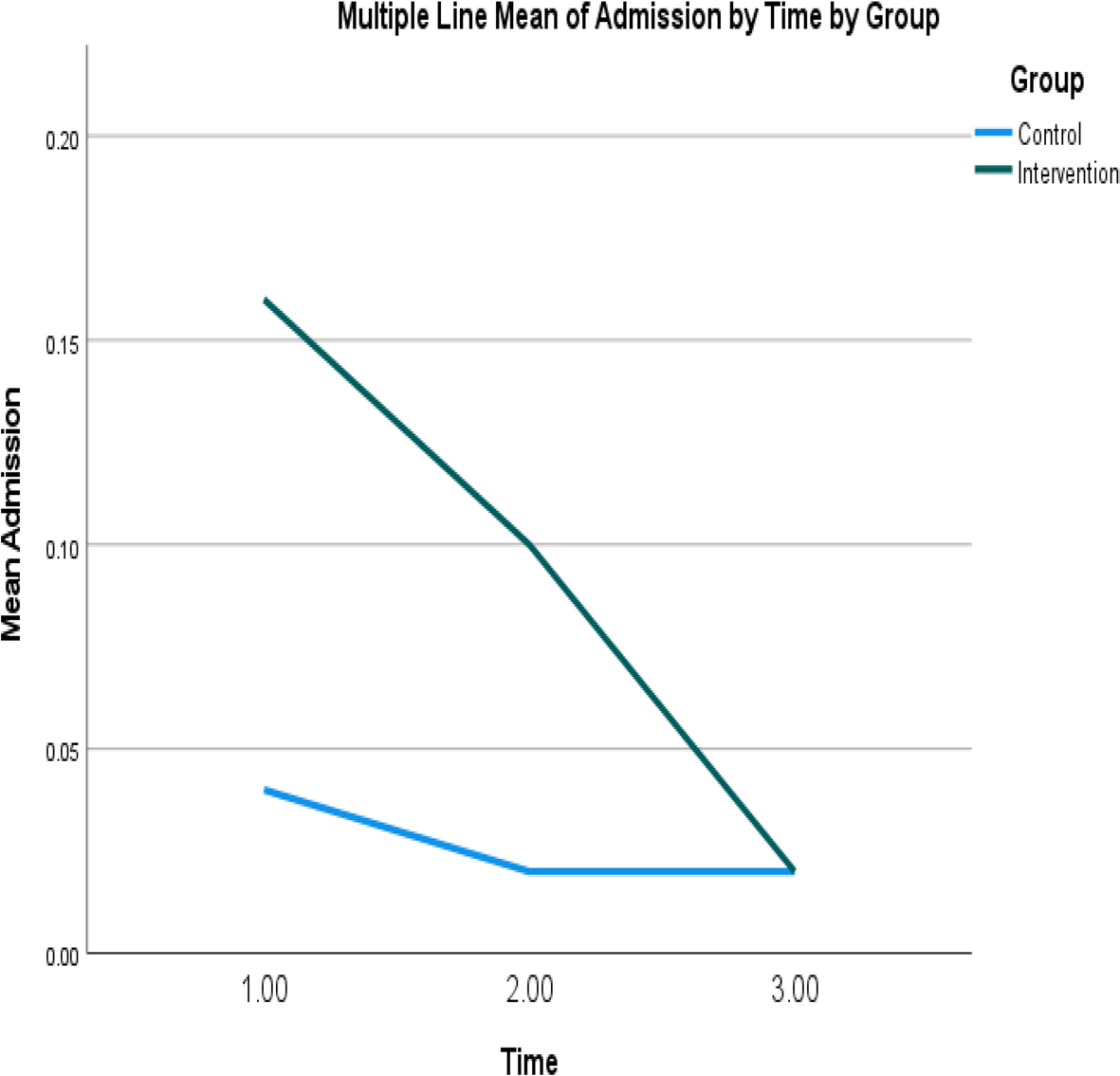
Mean of unplanned hospital admissions of intervention and control groups over time. Note: Higher mean scores indicate higher unplanned hospital admissions

The maintenance effects of the program were measured by evaluating the primary and secondary effectiveness outcomes 3 months after the completion of the program. No significant changes were observed from T1 and T2 to T3 in any outcome, although the numbers of unscheduled hospital admissions, unplanned general out-patient department visits, general practitioner visits, and emergency department visits, and the total number of health service uses, for the intervention group were lowest at T3 (Figs. 4, 5, 6, 7 and 8).

## Discussion

In recent years, researchers and policymakers around the world have emphasized the importance of successfully implementing health promotion programs in the real-world setting to improve public health. To do so, research must extend beyond evaluating the program’s effectiveness, to include the program’s reach indicators, adoption rate, implementation strategies, and sustainability. This translational study is one of the first to adopt a hybrid design in evaluating both the effectiveness and implementation outcomes of a community-based health-social partnership program. It demonstrated that the uptake of an HSPP for community-dwelling older adults could be successfully achieved with good reach, positive preliminary effectiveness, and high implementation fidelity in a non-governmental organization. These findings provide preliminary information on effective strategies for the implementation of self-care health management programs that can inform global policies and guide international health priorities to mitigate the chronic disease epidemic among community-dwelling older adults.

Despite difficulties in recruiting and engaging older adults into the program during the COVID-19 pandemic, the research team was able within 1 year to recruit a representative sample of community-dwelling older adults with similar demographic and clinical characteristics, including education level, chronic conditions, and number of health service uses. In addition, no differences were observed between older adults who participated in and completed the program and those who opted out. One reason for this high level of reach was the support of the community center staff during the recruitment process. The literature suggests that the recruitment of older adults is most successful when a person familiar to them promotes the program, such as the staff members of a community center that the older adults visit daily; these persons can arouse the interest of the older adults and assuage their concerns regarding participation in the program, thereby enhancing the recruitment rate and the reach level [47]. Furthermore, while the COVID-19 pandemic has reduced the sense of control among older adults, compromised their self-efficacy, and exacerbated a sense of victimization [48], it has also reminded them of the importance of health self-management [49]. Consequently, in addition to the active members who frequently join the health programs organized by community centers, the non-active members (i.e., those who seldom join community center programs) have started paying attention to their health and participating in the programs, which provides another explanation for the increased reach level of our program. A high reach level not only indicates the success of the logistics and promotion strategies of the program but also increases the generalizability and representativeness of the findings [38].

Our findings are consistent with studies that have identified several major barriers to widespread implementation of health programs among older adults, including manpower shortage, lack of experienced staff, and presence of an unpredictable and critical environment such as the COVID-19 pandemic. This study demonstrated that providing structured training workshops to the center staff, health and social care providers, and research assistants assigned to collect data or provide social calls can facilitate program implementation. The theoretical knowledge and practical skills covered in the workshops were necessary for the staff, providers, and research assistants to understand the purpose and content of the program, define roles of the team members, and conduct the interventions and necessary procedures in the program. A standardized training manual with a detailed protocol for the content compiled by the research team and validated by content experts can also be kept at the community center for use by new staff members tasked with program implementation; this can help sustain the program in the community over the long term. In addition, although the program was challenged by the COVID-19 pandemic, the research and service teams worked collaboratively according to a contingency plan to ensure that the program was successfully implemented for community-dwelling older adults in these critical times. Modifying the eligibility criteria to specifically include smartphone users, changing the delivery mode of providers from home visits to Zoom meetings, and providing IT support for both the providers and older adults are some contingency steps that can be deployed when face-to-face meetings with health care providers are not feasible. Our positive preliminary findings can guide policymakers and governments to allocate extra incentives and funding and designate additional manpower to non-governmental organizations to promote the health self-management of community-dwelling older adults.

A notable strength of this study is the inclusion of various key stakeholders in the design and implementation of a novel program that ensures the delivery of a well-rounded program of care. The process evaluation phase of the study provides further support for the preceding quantitative phase that helps to attain a greater explanatory power regarding the implementation of the program.

While the preliminary findings were encouraging, this study is not without limitations. First, this was a pilot study that included only one community center. Thus, although the reach level was high, this small study setting limits the generalizability of the findings. Second, due to the COVID-19 pandemic, the intended implementation strategies, such as home visits and data collection at the center, could not be carried out. Third, the study included only the older adults who own a smartphone and have Internet coverage at home. The findings may thus not be generalizable to those who have lower socioeconomic status and live in rural areas. While smartphone usage is more prevalent among older adults in these few years, future studies are recommended to include contingencies, such as lending smartphones to the older adults from the community center, receiving donations from smartphone companies, or replacing video call by phone call, to accommodate older adults who lack smartphones to avoid creating or perpetuating health disparities.

## Conclusion

This was a pioneer study to evaluate the translation of an HSPP in a community setting using a hybrid type-1 effectiveness–implementation design. The study fills a major gap in the literature on elderly care services by identifying strategies that facilitate effective implementation of older adult-targeted programs in the real-world setting. While there were barriers to and delays in the uptake of the program, the program showed reachability and preliminary effectiveness. The findings can inform the design and execution of health self-management programs and strategies for community-dwelling older adults.

## Supplementary Information

**Additional file 1.** Study flow of the programme

**Additional file 2.**

## Data Availability

The datasets generated and/or analysed during the current study are not publicly available due to a large dataset but are available from the corresponding author on reasonable request.

## Abbreviations

HSPP: Health–social partnership program
RE-AIM: Reach, effectiveness, adoption, implementation, and maintenance
HK-MoCA: Hong Kong version of Montreal Cognitive Assessment
NCM: Nurse case manager
CW: Community worker
SW: Social worker
TCM: Traditional Chinese medicine practitioner

## References

[1] Wong K, Yeung M. Population ageing trend of Hong Kong. Office of the Government Economist, The Government of the Hong Kong Special Administrative Region; 2019. p.14. Report No.: 2. https://www.hkeconomy.gov.hk/en/pdf/el/el-2019-02.pdf. Accessed 22 Apr 2022.

[2] Wu X, Law CK, Yip PSF (2019). A projection of future hospitalization needs in a rapidly ageing society: a Hong Kong experience. Int J Environ Res Public Health.

[3] Abdi S, Spann A, Borilovic J, de Witte L, Hawley M (2019). Understanding the care and support needs of older people: a scoping review and categorization using the WHO international classification of functioning, disability and health framework (ICF). BMC Geriatr.

[4] World Health Organization. Regional Office for South-East Asia. Self care for health: a handbook for community health workers and volunteers. WHO Regional Office for South-East Asia; 2014. https://apps.who.int/iris/handle/10665/205887.

[5] Orem DE (1995). Nursing: concepts of practice.

[6] Tiozzo SN, Basso C, Capodaglio G, Schievano E, Dotto M, Avossa F, Fedeli U, Corti MC (2019). Effectiveness of a community care management program for multimorbid elderly patients with heart failure in the Veneto region. Aging Clin Exp Res.

[7] Kim ES, Moored KD, Giasson HL, Smith J (2014). Satisfaction with aging and use of preventive health services. Prev Med.

[8] Alavijeh MS, Zandiyeh Z, Moeini M (2021). The effect of self-care self-efficacy program on life satisfaction of the Iranian elderly. J Educ Health Promot.

[9] Canjuga I, Zeleznik D, Neuberg M, Bozicevic M, Cikac T (2018). Does an impaired capacity for self-care impact the prevalence of social and emotional loneliness among elderly people?. Work Older People.

[10] Cramm JM, Nieboer AP (2017). Self-management abilities and quality of life among frail community-dwelling individuals: the role of community nurses in the Netherlands. Health Soc Care Community.

[11] Dye CJ, Willoughby DF, Battisto DG (2011). Advice from rural elders: what it takes to age in place. Educ Gerontol.

[12] Trask MA, Rozon C, Puyat JH, Costantini L, Mackay M, Ocampo LL, Marchuk S (2016). The evaluation of an orientation program of self-care abilities for patients on hemodialysis. Nephrol Nurs J.

[13] Hearld KR, Hearld LR, Budwani H, McCaughey D, Celaya LY, Hall AG (2019). The future state of patient engagement? Personal health information use, attitudes towards health, and health behavior. Health Serv Manag Res.

[14] Okpalauwaekwe U, Li CY, Tzeng HM (2022). Social determinants and self-care for making good treatment decisions and treatment participation in older adults: a cross-sectional survey study. Nurs Rep.

[15] Michie S, Atkins L, West R (2014). The behaviour change wheel: a guide to designing intervention.

[16] Schulz R, Beach SR, Czaja S (2020). Family caregiving for older adults. Annu Rev Psychol.

[17] Medical Research Council (2019). Developing and evaluating complex interventions: following considerable development in the field since 2006, MRC and NIHR have jointly commissioned an update of this guidance to be published in 2019.

[18] Funnell MM, Anderson RM, Arnold MS (1991). Empowerment: an idea whose time has come in diabetes education. Diabetes Educ.

[19] Bandura A (1977). Self-efficacy: toward a unifying theory of behavioural change. Psychol Rev.

[20] Wong AKC, Wong FKY, Yeung WF, Chang K (2018). The effect of complex interventions on supporting self-care among community-dwelling older adults: a systematic review and meta-analysis. Age Ageing.

[21] Burt J, Rick J, Blakeman T (2014). Care plans and care planning in long term conditions: a conceptual model. Prim Health Care Res Dev.

[22] Markle-Reid M, Browne G, Gafni A (2013). Nurse-led health promotion interventions improve quality of life in frail older home care clients: lessons learned from three randomized trials in Ontario, Canada. J Eval Clin Pract.

[23] Gustafsson S, Wilhelmson K, Eklund K, Gosman-Hedstrom G, Ziden L, Kronlof GH, Hojgaard B, Slinde F, Rothenberg E, Landahl S, Dahlin-Ivanoff S (2012). Health-promoting interventions for persons aged 80 and older are successful in the short term–results from the randomized and three-armed elderly persons in the risk zone study. J Am Geriatr Soc.

[24] Wong AKC, Wong FKY, Chang K (2019). Effectiveness of a community-based self-care promoting program for community-dwelling older adults: a randomized controlled trial. Age Ageing.

[25] Health Policy Project (2014). Capacity development resource guide: implementation barriers.

[26] Graziolo VS, Moullin JC, Kasztura M, Canepa-Allen M, Hugli O, Griffin J, Vu F, Hudon C, Jackson Y, Wolff H, Burnand B, Daeppen JB, Bodenmann P (2019). Implementing a case management intervention for frequent users of the emergency department (I-CaM): an effectiveness-implementation hybrid trial study protocol. BMC Health Serv Res.

[27] Yoong SL, Clinton-McHarg T, Wolfenden L (2015). Systematic reviews examining implementation of research into practice and impact on population health are needed. J Clin Epidemiol.

[28] Wolfenden L, Milat A, Lecathelinais C, Skelton E, Clinton-McHarg T, Williams CM, Wiggers J, Chai LK, Yoong SL (2016). A bibliographic review of public health dissemination and implementation research output and citation rates. Prev Med Rep.

[29] Bronfenbrenner U (1977). Toward an experimental ecology of human development. Am Psychol.

[30] Martin KS (2005). The Omaha system: a key to practice, documentation, and information management.

[31] Gittell JH, Weiss L (2004). Coordination networks within and across organizations: a multi-level framework. J Manag Stud.

[32] Yeung PY, Wong LL, Chan CC, Leung JLM, Yung CY (2014). A validation study of the Hong Kong version of Montreal cognitive assessment (HK-MoCA) in Chinese older adults in Hong Kong. Hong Kong Med J.

[33] Billingham SAM, Whitehead AL, Julious SA (2013). An audit of sample sizes for pilot and feasibility trials being undertaken in the United Kingdom registered in the United Kingdom clinical research network database. BMC Med Res Methodol.

[34] Chow SK, Wong FKY (2014). A randomized controlled trial of a nurse-led case management programme for hospital-discharged older adults with co-morbidities. J Adv Nurs.

[35] Bandura A (1997). Self-efficacy: the exercise of control.

[36] Durlak JA, DuPre EP (2008). Implementation matters: a review of research on the influence of implementation on program outcomes and the factors affecting implementation. Am J Community Psychol.

[37] The Chief Executive’s 2019 policy address. Treasure Hong Kong: our home. The government of the Hong Kong special administrative region; 2019. p. 33. https://www.policyaddress.gov.hk/2019/eng/pdf/PA2019.pdf. Accessed 27 Apr 2022.

[38] Glasgow RE, Vogt TM, Boles SM (1999). Evaluating the public health impact of health promotion interventions: the RE-AIM framework. Am J Public Health.

[39] Leung DYP, Leung AYM (2011). Factor structure and gender invariance of the Chinese general self-efficacy scale among soon-to-be-aged adults. J Adv Nurs.

[40] Lam CLK, Wong CKH, Lam ETP, Huang WW, Lo YYC (2010). Population norm of Chinese (HK) SF-12 health survey version 2 of Chinese adults in Hong Kong. Hong Kong Pract.

[41] Krueger RA, Casey MA (2015). Focus groups: a practical guide for applied research.

[42] Boyatzis RE (1998). Transforming qualitative information: thematic analysis and code development.

[43] Gale NK, Heath G, Cameron E, Rashid S, Redwood S (2013). Using the framework method for the analysis of qualitative data in multi-disciplinary health research. BMC Med Res Methodol.

[44] Lincoln YS, Guba EG (1986). But is it rigourous? Trustworthiness and authenticity in naturalistic evaluation. New Dir Eval.

[45] Marquart F. Methodological Rigor in Quantitative Research. In: Matthes J, Davis CS, Potter RF, editors. The International Encyclopedia of Communication Research Methods. 2017. 10.1002/9781118901731.iecrm0221.

[46] Census and Statistics Department. Thematic Household Survey Report no. 74. North Point, Hong Kong: Census and Statistics Department, Hong Kong Special Administrative Region; 2021. https://www.censtatd.gov.hk/en/data/stat_report/product/C0000092/att/B11302742021XXXXB0100.pdf. Accessed 2 May 2022

[47] Nichols EG, Shreffler-Grant J, Weinert C (2021). Where have they gone? Recruiting and retaining older rural research participants. Online J Rural Nurs Health Care.

[48] Falvo I, Zufferey MC, Albanese E, Fadda M (2021). Lived experiences of older adults during the first COVID-19 lockdown: a qualitative study. PLoS One.

[49] Fulmer T, Reuben DB, Auerbach J, Fick DM, Galambos C, Johnson KS (2021). Actualizing better health and health care for older adults. Health Aff.

