# Supplementary material for: A community-based health–social partnership program for community-dwelling older adults: a hybrid effectiveness–implementation pilot study
Source: BMC Geriatr. 2022 Oct 7;22:789. doi: 10.1186/s12877-022-03463-z (PMC9542442; doi:10.1186/s12877-022-03463-z)
Supplement: Supplementary file 1 — Additional file 1. Study flow of the programme [file 12877_2022_3463_MOESM1_ESM.docx]

Additional file 1 Study flow of the programme

Assessed for eligibility

Randomly assigned to intervention or control group

Intervention group Control group Data collection

Baseline pre-intervention (T1)

First month

First Zoom visit by nurse case manager at week 1

Telephone follow-up by nurse case manager at week 2

Telephone follow-up by community worker* at week 3

Social call at week 4

Telephone follow-up by community worker* at week 4

Second month

Social call at week 6

Zoom visit by the community worker * at week 6

Telephone follow-up by nurse case manager at week 8

Third month

Zoom visit by the community worker * at week 10

Program completed (T2)

Social call at week 12

Closing Zoom visit by the nurse case manager at week 12

*Supervised by nurse case manager

3 months after program (T3) completed (T3)
