# Supplementary material for: A community-based health–social partnership program for community-dwelling older adults: a hybrid effectiveness–implementation pilot study
Source: BMC Geriatr. 2022 Oct 7;22:789. doi: 10.1186/s12877-022-03463-z (PMC9542442; doi:10.1186/s12877-022-03463-z)
Supplement: Supplementary file 2 — Additional file 2. [file 12877_2022_3463_MOESM2_ESM.doc]

**Performance feedback form (NCM)**

Please tick in the appropriate box

□nurse case manager self-reflection

□Externally monitor session

| Items | Results | Remarks/ Comments |
| --- | --- | --- |
| Politeness (e.g. greeting, gesture) | - Satisfactory - Unsatisfactory - N/A |  |
| Assess the participant holistically by using Omaha system | - Satisfactory - Unsatisfactory - N/A |  |
| Set realistic contract goals with participant and formulate an individual care plan with participant | - Satisfactory - Unsatisfactory - N/A |  |
| Encourage the participant to maintain ongoing self-care behavior using bandura’s social theory | - Satisfactory - Unsatisfactory - N/A |  |
| Provide education according to their problems by using pamphlet | - Satisfactory - Unsatisfactory - N/A |  |
| Provide self-efficacy enhancing interventions | - Satisfactory - Unsatisfactory - N/A |  |
| Identify obstacles that may impede the participants to self-manage their own problems | - Satisfactory - Unsatisfactory - N/A |  |
| Think of strategies that can tackle these obstacles | - Satisfactory - Unsatisfactory - N/A |  |
| Provide and fill in the referral form according to the term-agreed guidelines and referral protocols whenever necessary | - Satisfactory - Unsatisfactory - N/A |  |
| Remind the next meeting schedule to the participant | - Satisfactory - Unsatisfactory - N/A |  |
| Review health and social goals with participant | - Satisfactory - Unsatisfactory - N/A |  |

**Performance feedback form (CW)**

Please tick in the appropriate box

□Community workers self-reflection

□Externally monitor session

| Items | Results | Remarks/ Comments |
| --- | --- | --- |
| Politeness (e.g. greeting, gesture) | - Satisfactory - Unsatisfactory - N/A |  |
| Support the NCM in monitoring the progress of the participants in accordance with their contract goals in Zoom meeting and telephone follow-up | - Satisfactory - Unsatisfactory - N/A |  |
| Provide social support in Zoom meeting and telephone follow-up | - Satisfactory - Unsatisfactory - N/A |  |
| Mobilizing community resources available in the district in Zoom meeting and telephone follow-up | - Satisfactory - Unsatisfactory - N/A |  |
| Arrange meals-on-wheel service when necessary | - Satisfactory - Unsatisfactory - N/A |  |
